# Supplementary material for: Shifts in protein aggregate stability define proteostasis decline in the aging human brain
Source: bioRxiv. 2026 Mar 30:2026.03.27.714902. Preprint. [Version 1] doi: 10.64898/2026.03.27.714902 (PMC13060232; doi:10.64898/2026.03.27.714902)
Supplement: Supplementary file 1 [file NIHPP2026.03.27.714902v1-supplement-1.pdf]

Supplementary Figure 1

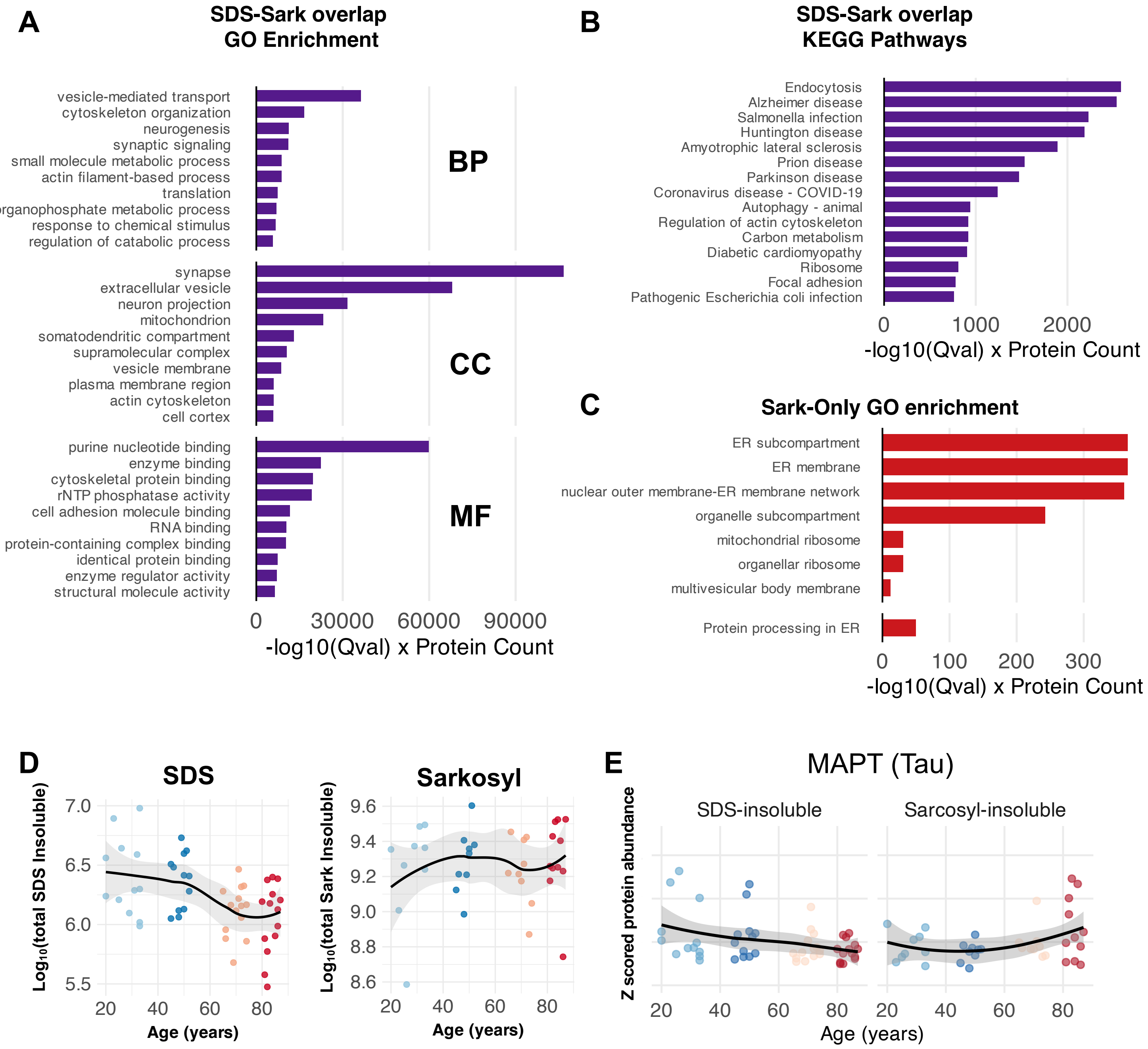

A. Barplot of top enriched GO terms for insoluble proteins common to both SDS and Sarkosyl fractions compared against the hippocampal proteome background, all q<0.05, Fisher's one-tailed test with Benjamini-Hochberg FDR correction. **B** As in (A) with top enriched KEGG pathway terms. **C.** Barplot of top enriched GO terms for insoluble proteins measured exclusively in the Sarkosyl fraction compared against the Sarkosyl insoluble detected as background, all q<0.05, Fisher's one-tailed test with Benjamini-Hochberg FDR correction. **D.** Loess plots of Log_{10}(Total Insoluble Signal) against subject age for SDS insoluble proteome (left) and Sarkosyl insoluble proteome (right), each data point represents an independent biological sample (individual donor). **E.** Loess plots of z-scored insoluble Tau protein abundance against subject age for SDS (left) and Sarkosyl (right), each data point represents an independent biological sample (individual donor).

Supplementary Figure 2

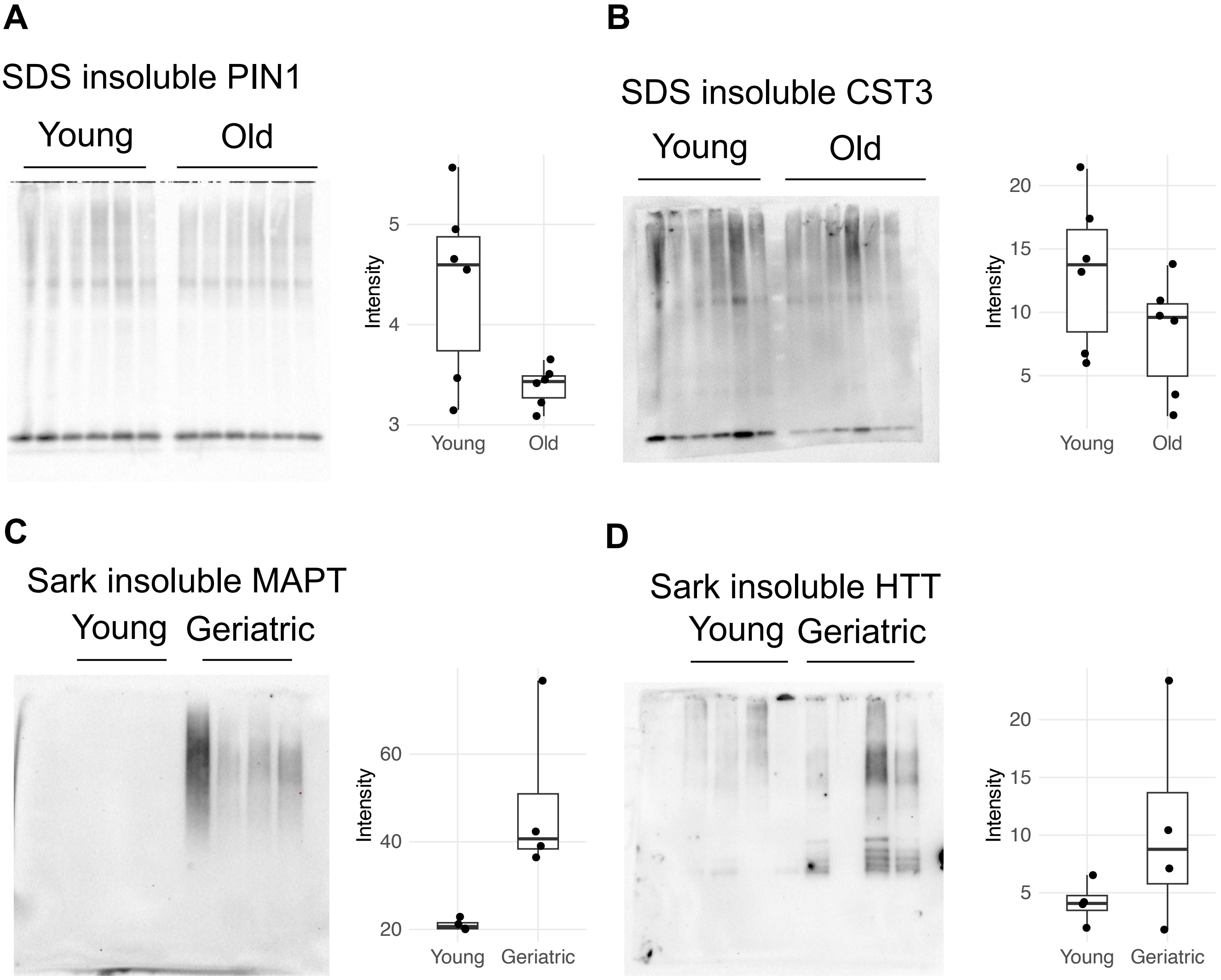

A. Western blot of SDS insoluble PIN1, 6 young and 6 old. **B**. Western blot of SDS insoluble CST3, 6 young and 6 old. **C**. Western blot of Sarkosyl insoluble MAPT (Tau), 4 young and 4 geriatric. **D**. Western blot of sarkosyl insoluble HTT, 4 young and 4 geriatric. For all blots each lane represents an independent biological sample (individual donor).

Supplementary Figure 3

A

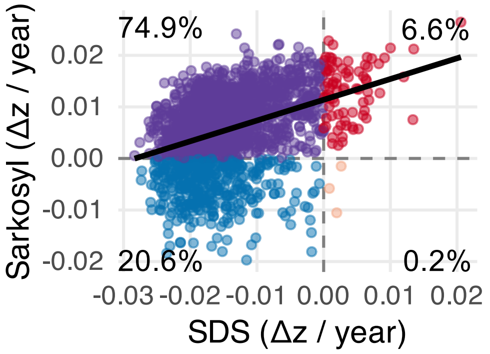

A Scatter plot for all proteins detected in both fractions showing Δz-score mean change over aging in the Sarkosyl fraction and SDS fraction. Spearman correlation line shows positive correlation.

Supplementary Figure 4

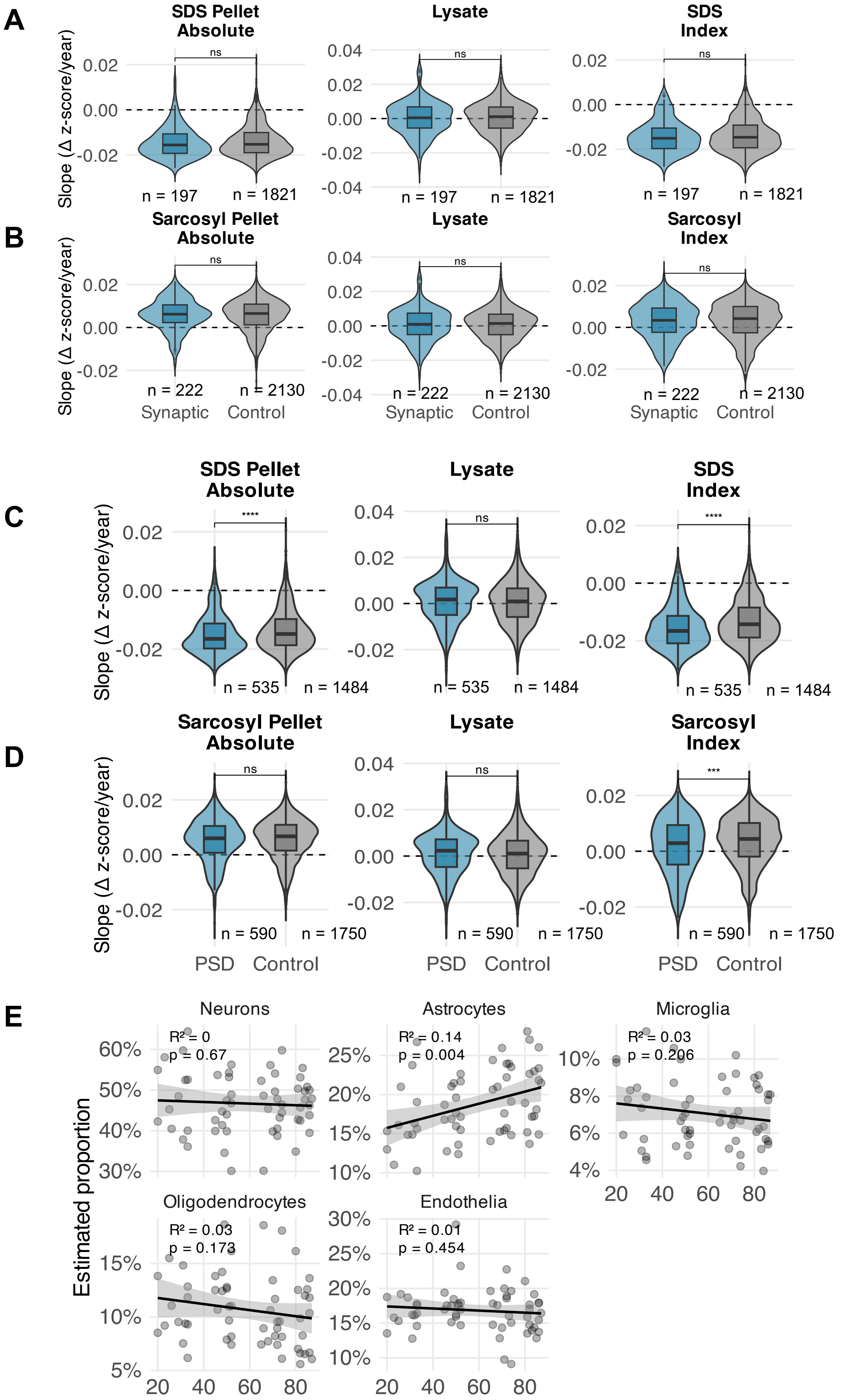

A Violin plot showing Δz-score mean change over aging for synaptic SDS insoluble proteins vs SDS insoluble detected proteins in terms of abundance in the pellet, abundance in the lysate, and the insolubility index (pellet / lysate), Mann Whitney U test. **B**. As in (A) with synaptic Sarkosyl insoluble proteins. **C-D**. As in (A-B) with post-synaptic density (PSD) insoluble proteins, Mann Whitney U test. **E.** Cell type proportion estimates for major central nervous system cell types. Pearson correlation analysis, estimated using dTangle and multibrain human reference signature, each data point represents an independent biological sample (individual donor).

Supplementary Figure 5

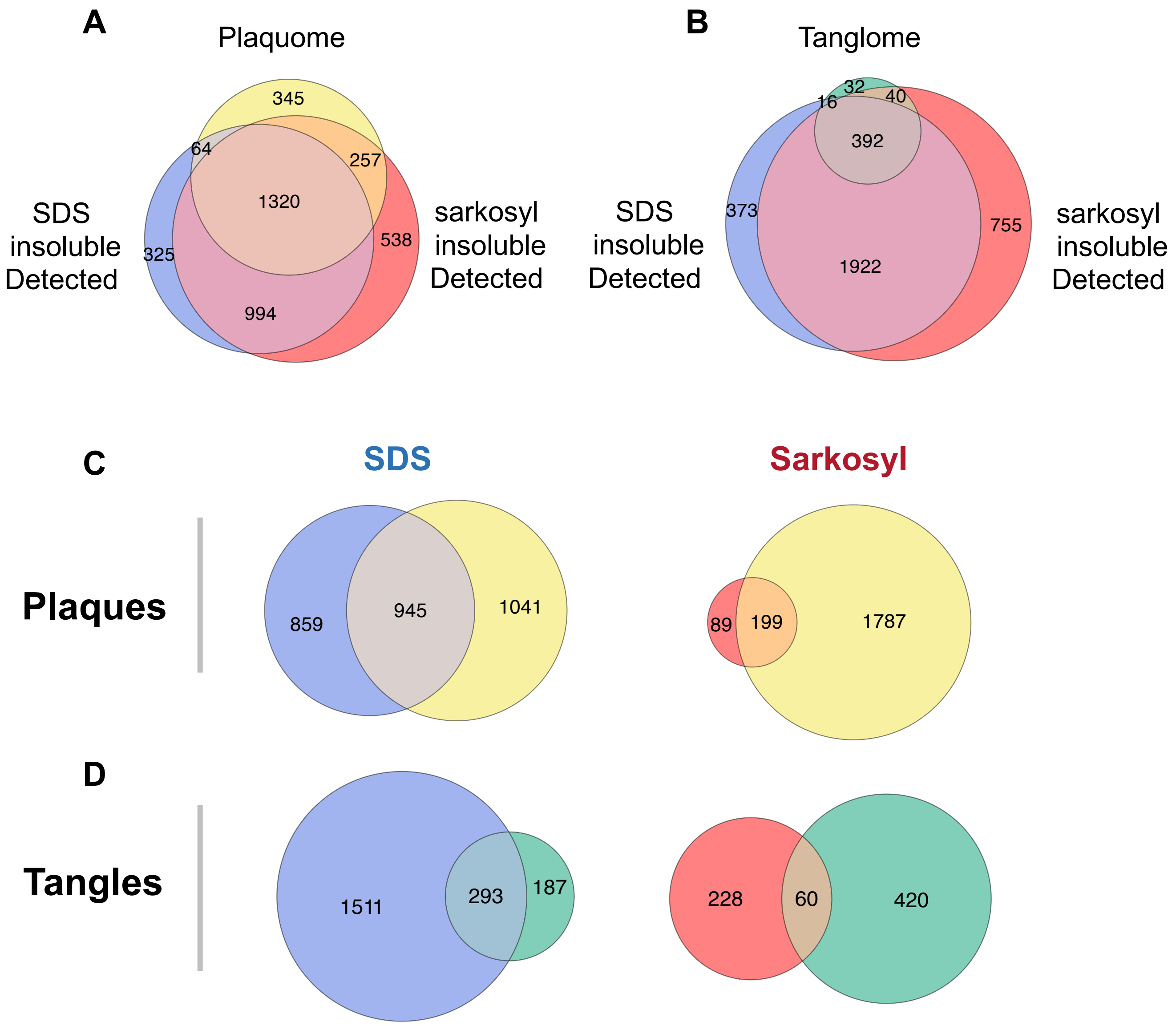

A Overlap between detected insoluble proteomes and plaquome. **B** Overlap between detected insoluble proteomes and tangleome. **C**. Overlap between age-changing proteins and plaqueome for SDS insoluble proteins (left) and Sarkosyl insoluble proteins (right). **D** As in © with tangleome for both fractions. Yellow = plaques, Green = Tangles, Blue = SDS, Red = Sarkosyl.

Supplementary Figure 6

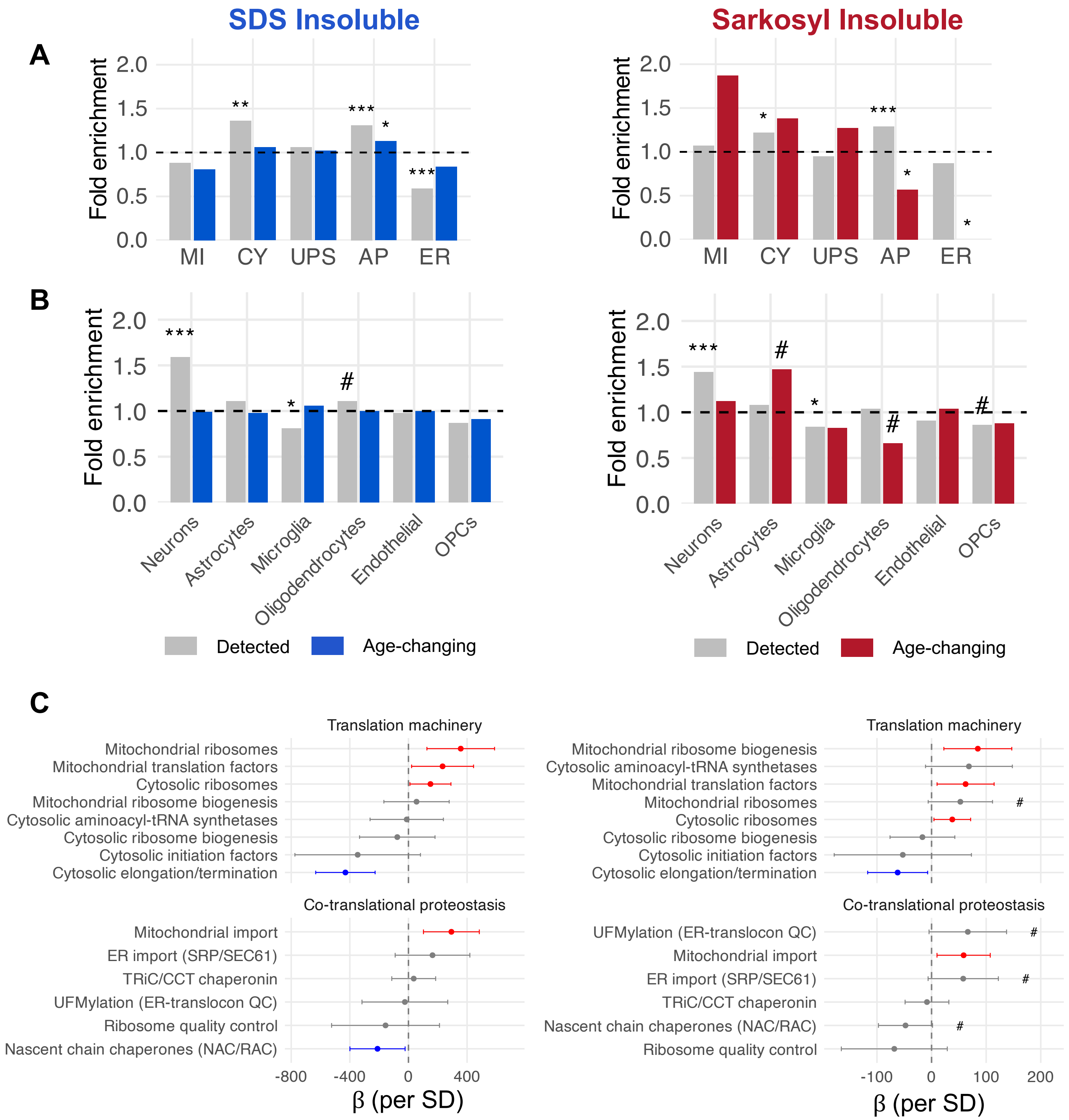

A Fold enrichment of proteostasis branch proteins in the insoluble proteome, Left = SDS, Right = Sarkosyl (Grey bars: insoluble detected, Blue: SDS age-changing, Red: Sarkosyl age-changing. **B** As in A, fold enrichment but using cell type signature scores and insoluble burden. Fisher's exact test with Benjamini-Hochberg correction. **C.** Forest plots showing β coefficient of age-adjusted linear models for translation-associated proteostasis network branch broken down into sub-branches left = SDS, right = Sarkosyl. Points show the beta coefficient +/- 95% confidence interval from a linear model adjusting for age (insol. burden ~ age + predictor). Red: significant positive ($p < 0.05$); Blue: significant negative ($p < 0.05$); grey, non-significant. # = p<0.1. MI = mitochondrial, CY = cytoplasmic, UPS = proteasomal, AP = autophagy. ***q < 0.001, **q < 0.01, *q < 0.05, #q <0.1. QC = Quality Control.
